# Behavioral constraints on local adaptation and counter‐gradient variation: Implications for climate change

**DOI:** 10.1002/ece3.6399

**Published:** 2020-06-17

**Authors:** Brandon M. Quinby, Mark C. Belk, J. Curtis Creighton

**Affiliations:** ^1^ Department of Biological Sciences Purdue University Northwest Hammond IN USA; ^2^ Department of Biology Brigham Young University Provo UT USA

**Keywords:** counter‐gradient variation, life history, local adaptation, *Nicrophorus orbicollis*, phenotypic variation

## Abstract

Resource allocation to growth, reproduction, and body maintenance varies within species along latitudinal gradients. Two hypotheses explaining this variation are local adaptation and counter‐gradient variation. The local adaptation hypothesis proposes that populations are adapted to local environmental conditions and are therefore less adapted to environmental conditions at other locations. The counter‐gradient variation hypothesis proposes that one population out performs others across an environmental gradient because its source location has greater selective pressure than other locations. Our study had two goals. First, we tested the local adaptation and counter‐gradient variation hypotheses by measuring effects of environmental temperature on phenotypic expression of reproductive traits in the burying beetle, *Nicrophorus orbicollis* Say, from three populations along a latitudinal gradient in a common garden experimental design. Second, we compared patterns of variation to evaluate whether traits covary or whether local adaptation of traits precludes adaptive responses by others. Across a latitudinal range, *N. orbicollis* exhibits variation in initiating reproduction and brood sizes. Consistent with local adaptation: (a) beetles were less likely to initiate breeding at extreme temperatures, especially when that temperature represents their source range; (b) once beetles initiate reproduction, source populations produce relatively larger broods at temperatures consistent with their local environment. Consistent with counter‐gradient variation, lower latitude populations were more successful at producing offspring at lower temperatures. We found no evidence for adaptive variation in other adult or offspring performance traits. This suite of traits does not appear to coevolve along the latitudinal gradient. Rather, response to selection to breed within a narrow temperature range may preclude selection on other traits. Our study highlights that *N. orbicollis* uses temperature as an environmental cue to determine whether to initiate reproduction, providing insight into how behavior is modified to avoid costly reproductive attempts. Furthermore, our results suggest a temperature constraint that shapes reproductive behavior.

## Graphical abstract

Our study provides unique insight into how behavior is modified to avoid costly reproductive attempts. Our results suggest a temperature tolerance constraint that shapes reproductive behavior and distribution. This suite of reproductive traits does not appear to coevolve with the latitudinal gradient in a coordinated fashion.

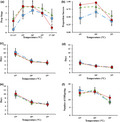

## 1 INTRODUCTION

Life history patterns are the result of an organism's genotype and its interaction with the environment (Stearns, 2015; Yamahira & Conover, 2002). As such, life histories are expected to vary phenotypically along environmental gradients. Environmental temperature variation along latitudinal gradients has been linked specifically to varying growth rates in fishes (L'Abee‐Lund et al., 1989; Trip, Clements, Raubenheimer, & Choat, 2014) and longer development times, leading to larger body size, of reptiles and insects (Laiolo & Obeso, 2015; Morrison & Hero, 2003). Temperature variation can also affect breeding success. For example, temperature extremes decrease egg hatchability in the corn leafhopper *Dalbulus maidis* (Van Nieuwenhove, Frías, & Virla, 2016) and result in reduced fecundity and egg and larval survival in the bagworm *Thyridopteryx ephemeraeformis* (Lynch et al., 2014).

Variation in trait expression along environmental gradients can be the result of different selective pressures driving diversification among populations. If we represent an environmental gradient as only the two extreme end points (e.g., Conover & Schultz, 1995), then we do not allow different selective processes to be observed in our design, or our result is a confounding of multiple selective processes. By adding intermediate levels in our sampling and experimental design, we can more fully explore the potential complexity of phenotypic expression along large‐scale environmental gradients.

**FIGURE 1:**
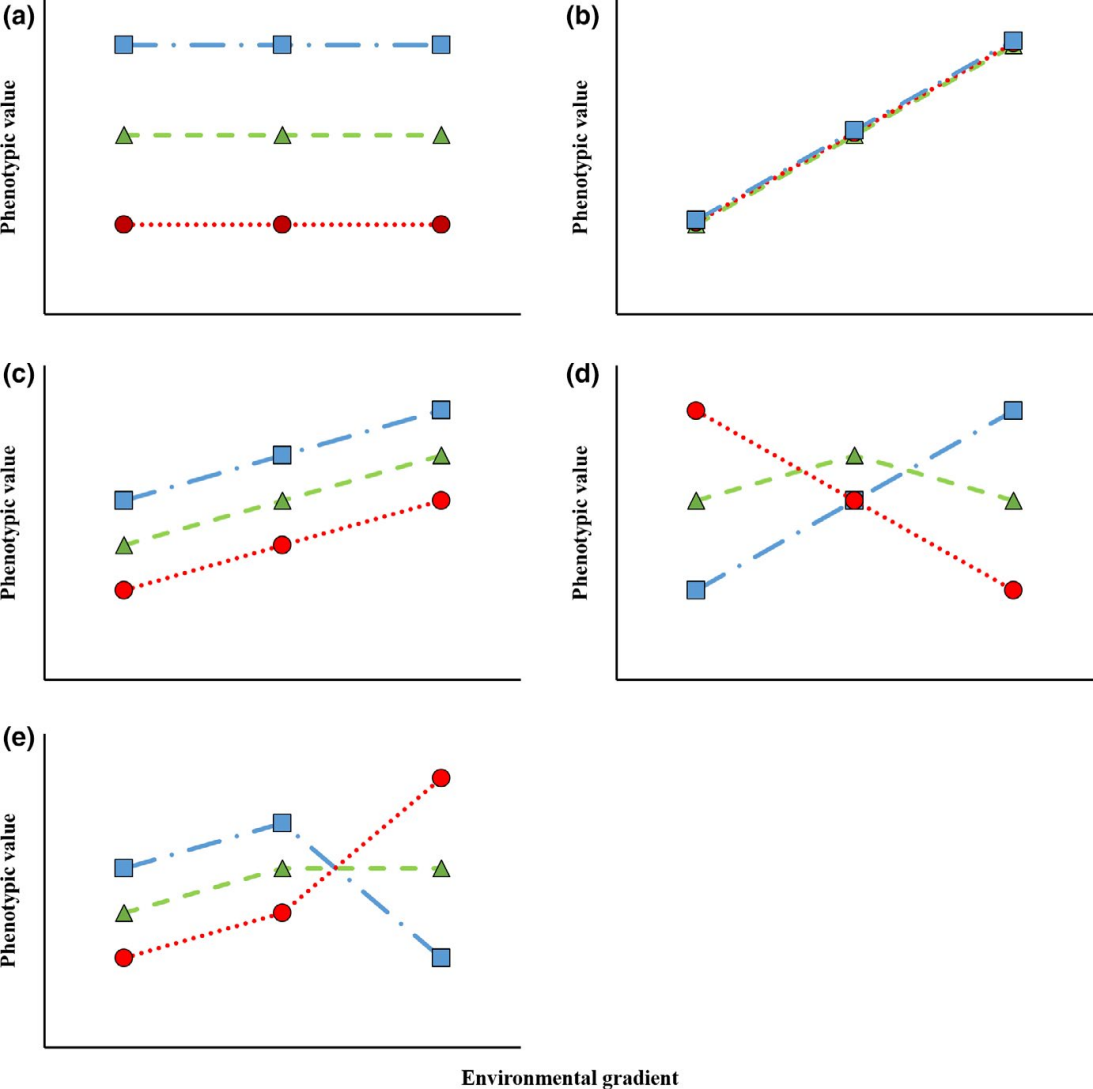
Figure illustrating the predicted results of expressed phenotypic values for each of the tested hypotheses across an environmental gradient for a high latitude 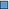, medium latitude 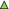, and low latitude 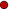 population. (a) No plasticity in phenotypic values. (b) No genetic variation in phenotypic values. (c) Co/counter‐gradient variation in phenotypic values. (d) Local adaptation in phenotypic values. (e) Co/counter‐gradient variation and local adaptation in phenotypic values

A powerful way to evaluate genetic and environmental effects on life history variation is with a common garden experiment. This approach, where individuals originating from different populations are raised under controlled environmental conditions, allows researchers to tease apart genetic and environmental contributions to phenotypic variation (Conover & Schultz, 1995). Phenotypic variation, measured across an environmental gradient, is referred to as a reaction norm (see reviews by Angilletta, 2009; Gotthard & Nylin, 1995; Schlichting & Pigliucci, 1998; Stearns, 1989; West‐Eberhard, 2003). In the most straight forward results from this type of experiment, there may be no environmental effect (Figure 1a) or no genetic variation (Figure 1b) for the traits of interest. Alternatively, the tested populations can show counter‐gradient or cogradient variation (Figure 1c). Under these circumstances, there is no trade‐off in the trait examined between adaptive performance in one environment and performance in another (Conover & Schultz, 1995). Finally, a population may show local adaptation where each tested population performs best in conditions most similar to its native environment (Figure 1d). Neither of the covariation hypotheses are mutually exclusive with local adaptation across a broad environmental gradient (Figure 1e) with more than two levels of the environmental gradient represented in the common garden experiment.

How does selection create these alternative patterns of variation? Counter‐gradient variation arises when environmental pressures impede the development of phenotypically plastic traits, and selection increases investment on the trait to counteract negative environmental effects. Because counter‐gradient variation counteracts the effect of the local environment, the result is less latitudinal variation in phenotypes than expected (Conover & Schultz, 1995; Laiolo & Obeso, 2015; Levins, 1968). Counter‐gradient variation manifests as higher trait expression by populations originating in the more stressful environment in both native and novel environments in common garden experiments. In contrast, cogradient variation occurs when populations evolving under favorable environmental conditions exhibit increased trait expression across all environments (Conover, Duffy, & Hice, 2009). Because cogradient variation acts in the same direction as the environmental potential for trait expression, cogradient variation enhances phenotypic variation across the environmental gradient. Local adaptation occurs when each population is adapted to the conditions unique to its local range, resulting in each population performing best at conditions most similar to their local environment (Kawecki & Ebert, 2004). Thus, traits measured from different populations across an environmental gradient show a significant interaction effect, represented by crossing reaction norms (Stearns, 2015; Stearns & Koella, 1986).

Reproductive strategy consists of a suite of coevolved traits (Endler, 1995; Parsons & Joern, 2014; Stearns, 1992). Thus, two types of questions arise in an evaluation of effects of latitudinal gradients. First, is the question of whether an individual reproductive trait conforms to the pattern of local adaptation or counter‐gradient variation? Much like the assessment of growth rate in several other studies (Ayres & Scriber, 1994; Niewiarowski & Roosenburg, 1993), this question views evolution of a given trait as unconstrained by the evolution of other traits. Second, is the question of whether the multiple traits covary or whether their response is coordinated in some way? For example, if one trait exhibits a pattern consistent with local adaptation, do all traits show this same pattern? Do reproductive traits evolve in a coordinated fashion in response to latitudinal gradients as they have been shown to do in response to predation or resource availability (Balasubramaniam & Rotenberry, 2016; King, Roff, & Fairbairn, 2011; Reznick & Endler, 1982)? Compared to growth, reproduction is a complex activity that involves several traits and processes. It is unclear how these traits and processes vary across a latitudinal gradient and whether adaptive variation in some traits precludes or mitigates adaptive responses in other related traits (Donelson, Salinas, Munday, & Shama, 2018; Huey, Hertz, & Sinervo, 2003). Furthermore, there are few experiments accounting for parental effects on adaptation hypothesis (Badyaev & Uller, 2009; Fox, Nilsson, & Mousseau, 1997; Hutchings, 2011; Uller, 2008).

In this paper, we evaluate these hypotheses relative to a suite of traits associated with reproduction in the burying beetle, *Nicrophorus orbicollis*, a species that ranges from the southeastern United States into southeastern Canada and west to Saskatchewan (Anderson, 1982). Burying beetles (Silphidae: *Nicrophorus*) reproduce on small vertebrate carcasses, which are the sole source of food for offspring and parents during larval development, making control and assessment of resources quantifiable (Creighton, 2005; Eggert & Müller, 1997; Scott, 1998; Trumbo, 1990). Parents regulate brood size through filial cannibalism resulting in an increase in brood size with increasing carcass size (Bartlett, 1987; Creighton, 2005). Adult body size is an important determinant of competition for carcasses with the largest male and female arriving at the carcass gaining possession (Eggert & Müller, 1997; Scott, 1998).

Burying beetles are unusual among insects in that they provide prehatching and posthatching biparental care. Prehatching care includes burying the carcass and preserving it by removing fur or feathers, rolling it into a ball, and applying antimicrobial secretions (a form of social immunity; Cotter & Kilner, 2010; Hoback, Bishop, Kroemer, Scalzitti, & Shaffer, 2005). After larvae begin to arrive on the carcass to feed, parental care continues with the creation of a small feeding hole in the carcass, regurgitation of carrion directly to the larvae, and defense of the young from predators (Eggert & Müller, 1997; Scott, 1998). In *N. orbicollis*, the young are dependent on parental care until the third instar stage when they begin to rely on self‐feeding until dispersal into the soil where they pupate (Scott, 1998).

Reproduction in *N. orbicollis* is costly. Females that do not reproduce live significantly longer than females that reproduce, and females reproducing on larger carcasses die faster than females reproducing on smaller carcasses (Billman, Creighton, & Belk, 2014; Creighton, Heflin, & Belk, 2009). When females reproduce multiple times, each subsequent reproductive attempt results in a decrease in the number of offspring produced (Billman et al., 2014; Creighton et al., 2009). The investment in social immunity by the parents is also very costly, and increased social immunity investment results in decreased fitness for the parents (Cotter, Topham, Price, & Kilner, 2010).

In this study, we evaluate reproductive strategies in *N. orbicollis* across a temperature gradient using three latitudinally distinct beetle populations. First, we tested the local adaptation and counter‐gradient variation hypotheses by quantifying measures of (a) parental reproductive performance including reproductive success, hatching asynchrony, offspring number, and developmental timelines; and (b) offspring performance including growth rate, adult body size, offspring developmental stability (as measured by degree of fluctuating asymmetry of newly eclosed adults), and percent body fat. We evaluated these traits using a common garden design at multiple temperatures. The local adaptation hypothesis predicts that each population will perform best at the temperature that best approximates their native location. The result would be crossing reaction norms. In contrast, the counter‐gradient variation hypothesis predicts that one population will perform best at all temperatures. Second, we compared patterns of variation among traits to evaluate whether these traits covary across the temperature gradient or whether local adaptation of some traits precludes adaptive responses by others. For example, initiation of reproduction across the temperature gradient may show local adaptation, but other traits may not differ among populations because strong selection on whether to reproduce at a given temperature may limit selection on traits that occur afterward.

If low temperatures constrain carcass preparation and reproduction, then counter‐gradient variation would be manifest as greater success and less asynchrony from populations from higher latitudes across all temperatures. Conversely, if high temperatures constrain carcass preparation and reproduction, then counter‐gradient variation would be manifest as greater success and less asynchrony from populations from lower latitudes across all temperatures. Local adaptation would be manifest as crossing reaction norms with each population performing best at the temperature that most closely approximates the mean temperature during the breeding season in their native location.

Assuming that minimizing developmental times leads to increased fitness (e.g., better survival of offspring from egg to adulthood), counter‐gradient variation in developmental time would be manifest as shorter development times in one population relative to the others. Local adaptation would be manifest as crossing reaction norms with shorter development times in populations at the temperature that most closely approximates the temperature in their native location. Additionally, counter‐gradient variation would be manifest as more surviving offspring by one population compared to the other two across all temperatures. Local adaptation would be manifest as a crossing reaction norm with each population producing more offspring at the temperature that most closely approximates the temperature in their native location.

Similar to our previous measures with adult beetles, if low temperatures constrain developmental stability for larvae, then counter‐gradient variation would be manifest as greater stability from populations from higher latitudes across all temperatures, that is, faster growth rates, larger adult offspring body size, lower levels of fluctuating asymmetry, and higher percentage body fat. Conversely, if high temperatures constrain developmental stability for larvae, then counter‐gradient variation would be manifest as greater success from populations from lower latitudes across all temperatures. Local adaptation would be manifest as crossing reaction norms with each population performing best at the temperature that most closely approximates the mean temperature during the breeding season in their native location.

## 2 MATERIALS AND METHODS

### 2.1 *Nicrophorus orbicollis* populations

**FIGURE 2:**
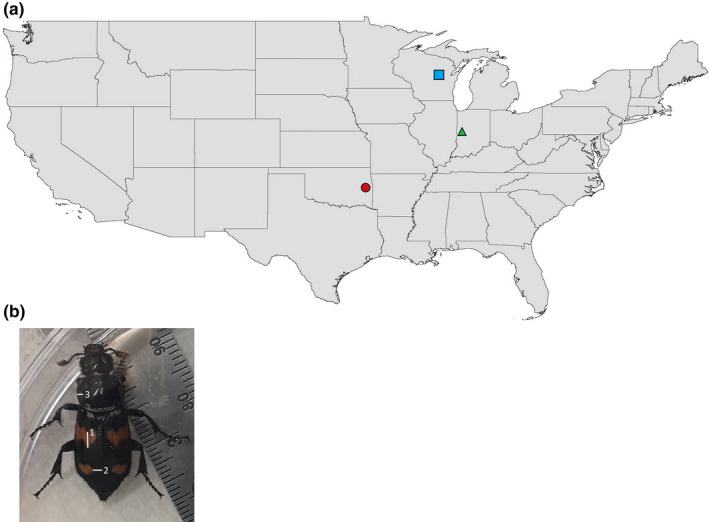
(a) Beetles for experiments were derived from wild‐caught individuals collected in Big Falls, Wisconsin; (high latitude 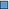; latitude 44.6165°N, longitude −89.0161°W); Waveland, Indiana; (medium latitude 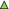; latitude 39.9417°N, longitude –87.0917°W); and Spavinaw, Oklahoma; (low latitude 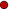; latitude 35.3704°N, longitude −95.0486°W). We brought wild‐caught beetles into the laboratory to procure the F1 generation of beetles used for experiments. (b) Schematic on fluctuating asymmetry measurements. We measured three variables: (1) an anterior to posterior transect through the beetles upper orange elytra spot, (2) a basal to distal transect from the elytra edge to the lower orange elytra spot, and (3) a basal to distal transect from the “pronotum cleft” to the edge of the pronotum, on both the left and right sides of each individual beetle

We derived the laboratory beetle populations used for this experiment from wild‐caught beetles captured with baited pitfall traps near Big Falls, Wisconsin (high latitude; HL; 44.6165°N, −89.0161°W), Waveland, Indiana (mid latitude; ML; 39.9417°N, −87.0917°W), and Spavinaw, Oklahoma (low latitude; LL; 35.3704°N, −95.0486°W) (Figure 2a) in May–June of 2014 and 2015. We housed all *N. orbicollis* in individually marked plastic containers (15 × 11 × 7 cm) in an environmental chamber at 21°C with a 14:10 hr light:dark (L:D) cycle and fed chicken liver ad libitum. These conditions simulated the natural light/dark pattern and temperature consistent with the beetles’ summer breeding season in their natural environment (Cook, Smith, Meyers, Creighton, & Belk, 2019). We used these wild‐caught beetles to establish the first generation (F1) laboratory populations used for experiments. We bred wild‐caught beetles by placing a male and female with a fresh mouse carcass in plastic containers (18 × 15 × 10 cm) filled two‐thirds full with topsoil. We removed the wild‐caught males when larvae first appeared on the carcass, and we removed wild‐caught females when larvae dispersed from the carcass. We left F1 larvae undisturbed until eclosion (approximately 28–30 days), and then, we maintained them in individual plastic containers as described above until used for the experiments at sexual maturity (21‐ to 28‐day‐old posteclosion).

### 2.2 Common garden experiment

**FIGURE 3:**
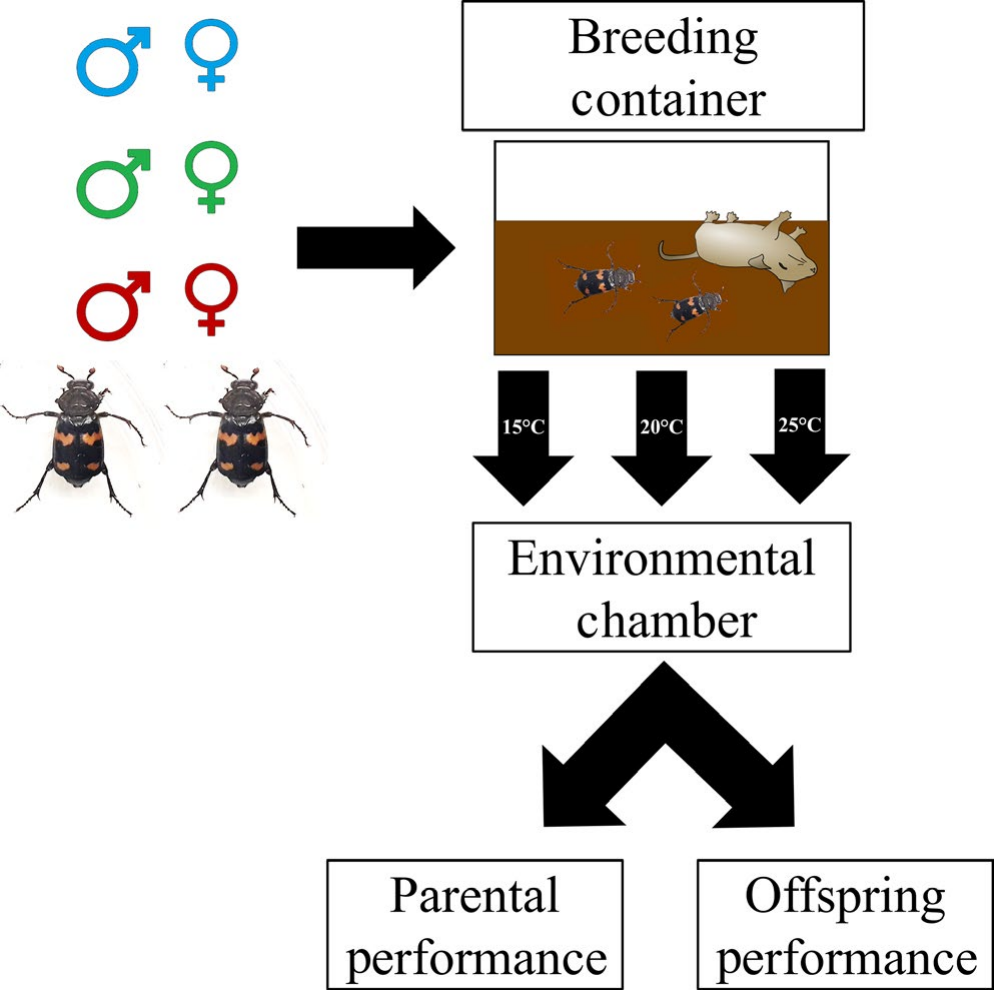
Schematic of experimental design. We randomly selected sexually mature (21–28 days old) male and females from laboratory‐reared F1 Generation beetles from three populations (high latitude—blue; medium latitude—green; and low latitude—red), and placed them along with a fresh mouse carcass in a plastic breeding container (18 × 15 × 10 cm) filled two‐thirds full with topsoil. We placed breeding containers in an environmental chamber maintained at each of five constant air temperatures (12–13°C, 15°C, 20°C, 25°C, or 27–28°C) For schematic simplicity, and because beetle pairs from all populations were not successful at rearing larvae at *T*_min_ (12–13°C) or *T*_max_ (27–28°C), they are not included in the figure. We checked, photographed, and monitored broods daily to measure response variables. We monitored all broods until beetles completed the reproductive cycle or until we determined brood failure (i.e., carcass preparation stopped). To measure parental performance, we evaluated the following variables: reproductive success, hatching asynchrony, offspring number, and developmental timelines. To measure offspring performance, we evaluated the following variables: offspring growth rate, adult offspring body size, developmental stability (as measured by degree of fluctuating asymmetry of newly eclosed adults), and percentage offspring body fat

To assess genetically based latitudinal patterns, we tested representatives from each of the three source locations at each of five constant air temperatures (12–13°C, 15°C, 20°C, 25°C, or 27–28°C) in a common garden experimental design. We began each trial by randomly selecting a laboratory‐reared, sexually mature (21–28 days old) male and female from the same population, and from different parental lines. At the beginning of each reproductive bout, we weighed females and males and measured their pronotum width. We placed each pair of beetles in a plastic container (18 × 15 × 10 cm) filled two‐thirds full with commercially purchased topsoil and given a freshly thawed 30 g (± 1 g) mouse carcass (Figure 3). Because either parent can successfully raise a brood if their partner is handicapped or removed (Creighton, Smith, Komendat, & Belk, 2015; Smiseth, Dawson, Varley, & Moore, 2005), we removed males after 48 hr to allow sufficient time for mating to occur, but to minimize male impact on female life history characteristics thereafter (Creighton et al., 2015; Rauter & Moore, 2004; Smiseth et al., 2005; Smith, Creighton, & Belk, 2015). We randomly assigned pairs from each of the three populations to a treatment of one of five temperatures: 12–13°C (*T*_min_), 15°C (average three‐month daily low temperature for Wisconsin), 20°C (average three‐month daily temperature for Indiana), 25°C (average three‐month daily high temperature for Oklahoma), or 27–28°C (*T*_max_); (L:D;14:10; Figure 3). Temperatures represent the average daily high and low temperatures experienced within the range of the collection sites for the known breeding months of May–August. We determined experimental breeding temperatures by calculating a three‐month daily mean air temperature for each location using ten‐year temperature data sets obtained from the National Climatic Data Center (NCDC) Annual Climatological Summaries of U.S. station data (ncdc.noaa.gov accessed on 9/10/2013). We checked, photographed, and monitored broods daily to measure response variables. We monitored all broods until beetles completed the reproductive cycle or until we determined brood failure (i.e., carcass preparation stopped). Each temperature treatment by location combination had at least 10 replicates, but more broods were required at the intermediate temperatures to allow for analysis of offspring traits. We summarized sample sizes for each temperature treatment by location combination in Table 1.

**TABLE 1:** Summary of sample sizes (*N*) for locations (HL, ML, and LL) at temperatures (12–13°C, 15°C, 20°C, 25°C, and 27–28°C)

Temperature (°C)	Locations		
	HL	ML	LL
12–13°	10	10	10
15°	25	26	25
20°	37	31	29
25°	35	38	36
27–28°	18	17	16

### 2.3 Parental reproductive performance

We measured both parental and offspring response variables from each brood to characterize variation in reproductive performance across the temperature gradient. To measure parental performance, we evaluated the following variables: reproductive success, hatching asynchrony, offspring number, and developmental timelines. Each of these response variables yielded one measure per brood, so sample sizes are equivalent to those in Table 1 for the three intermediate temperatures.

#### 2.3.1 Carcass preparation and reproductive success

We measured reproductive success by two response variables—degree of carcass preparation and probability of producing offspring that survived to adulthood. We scored carcasses for degree of preparation and assigned a score of zero to four to represent the stage of carcass preparation (Table 2). We evaluated the final stage of carcass preparation for each brood as a measure of reproductive success at different temperatures. To characterize the probability of producing offspring at a given temperature, we scored each brood as a success (score = 1) if any adult offspring were produced, or a failure (score = 0) if no adult offspring were produced.

**TABLE 2:** Definitions of stages of carcass preparation. Beetle pairs were observed, photographed, and scored daily during the carcass preparation stage and were assigned a number 0–4 based upon the appearance of the carcass

Score	Stage of preparation
0	Carcass not prepared or abandoned/Female found feeding on carcass
1	Carcass preparation started ≤25% of hair removed; carcass not balled up; head, legs, and tail of mouse still distinguishable
2	˃25% ≤50% of hair removed; carcass partly balled ≤50%; carcass still mouse like in appearance but mouse characteristics such as head and legs; partially distinguishable
3	˃50% ≤75% of hair removed; carcass mostly balled ≤75%; mouse characteristics of carcass mostly absent except tail and head
4	Carcass fully prepared 100% of hair removed; carcass fully balled up; carcass looks like a mummified ball and all mouse characteristics are absent; feeding hole present

#### 2.3.2 Hatching asynchrony

We calculated brood hatching asynchrony as the spread of hatching dates within a brood (i.e., the number of days between the first and last arriving first instar larvae on the prepared carcass; Aparicio, 1999). While there may be an adaptive value to increased asynchrony, for this analysis we assumed that less asynchrony in larval arrival to a carcass is advantageous.

#### 2.3.3 Parental developmental timelines and reproductive output

To determine parental developmental timelines, we used two variables: time in days: (a) to fully prepare the carcass; and (b) for larvae to fully develop and consume the carcass (Scott, 1998). To characterize reproductive output, or number of offspring produced, we determined the number of final (3rd) instar larvae that dispersed from the carcass.

### 2.4 Offspring performance

To measure offspring performance, we evaluated the following variables: offspring growth rate, adult offspring body size, developmental stability (as measured by degree of fluctuating asymmetry of newly eclosed adults), and percentage offspring body fat.

#### 2.4.1 Offspring growth rate and brood size

To evaluate offspring growth rate, we subtracted the average mass of an individual larvae from the first day they were present on the carcass from the average mass of an individual larvae on the final day they were present on the carcass. We then divided this value by the total number of days that larvae were on the carcass to account for asynchronous arrival and dispersal to the carcass by larvae. The response variable was mean offspring growth rate per brood. To measure offspring body size, we measured the adult offspring body mass from all offspring in the brood at the time of eclosion. We used mean offspring body size as the response variable for analysis.

#### 2.4.2 Offspring developmental stability

To measure developmental stability and percentage body fat, we randomly selected one adult male and one adult female offspring from each brood. This selection resulted in two replicates of developmental stability and percent body fat per brood for analysis. These individuals were pinned for the purpose of taking photographs which were then used to measure fluctuating asymmetry. We used three variables: (a) an anterior to posterior transect through the beetles upper orange elytra spot, (b) a basal to distal transect from the elytra edge to the lower orange elytra spot, and (c) a basal to distal transect from the “pronotum cleft” to the edge of the pronotum (Figure 2b). We used the difference between the left and right side for each of these variables as a measurement of developmental stability (Van Valen, 1962). Additionally, we determined percentage body fat on these same individuals following the lipid extraction techniques used by Marden (1989).

### 2.5 Data analysis

No beetles produced offspring at either the highest or lowest of the five temperatures in the experimental design. For this reason, only degree of carcass preparation was analyzed at all five temperatures. Because the response variable was discrete (i.e., scored as an integer from 0 to 4), we used a generalized linear model with a log link function and a Poisson distribution (Neter, Wasserman, & Kutner, 2004). The model had two fixed factors: temperature treatment (5 levels) and location (3 levels). We included the interaction between temperature treatment and location, and female and male body size as covariates. We used Proc GENMOD in SAS for the analysis (SAS 9.3 SAS Institute).

We modeled the probability of producing offspring (i.e., reproductive success) as a binomial response (0 or 1) with a probit link function in a generalized linear model framework with a Poisson distribution (Neter et al., 2004). Because no beetles produced offspring at the highest and lowest temperatures in the experimental design, we used only the three intermediate temperatures for this analysis. The model had two fixed factors: temperature treatment (3 levels) and location (3 levels). We included the interaction between temperature treatment and location, and female and male body size as covariates. We used Proc GENMOD in SAS for the analysis (SAS 9.3 SAS Institute).

A number of offspring produced (i.e., final brood size), hatching asynchrony, and developmental timing (2 response variables) were represented as count data, so we used a generalized linear model with a log link function and a Poisson distribution (Neter et al., 2004). The model had two fixed factors: temperature treatment (3 levels) and location (3 levels), and we included the interaction between temperature treatment and location. For number of offspring produced, time preparing the carcass, and time until dispersal of offspring, we included female and male body size as covariates. For the model of hatching asynchrony, we included female mass and brood size as covariates. We used Proc GENMOD in SAS for each of these analyses (SAS 9.3 SAS Institute).

Growth rate of offspring and size of adult offspring (measured as mass in g) were continuous response variables so we used a general linear model and a Poisson distribution (Neter et al., 2004) on untransformed data for analysis. Data consisted of means calculated for each brood so we have one replicate per brood. Raw data exhibited normally distributed residuals and fit the assumptions of the model. The model had two fixed factors: temperature treatment (3 levels) and location (3 levels), and we included the interaction between temperature treatment and location. For the growth rate model, we included female combined parental body size and brood size as covariates, and for the offspring size model, we included female body size, male body size, and brood size as covariates. We used Proc MIXED in SAS for the analysis (SAS 9.3 SAS Institute).

To evaluate percent body fat and developmental stability (three measures of fluctuating asymmetry; S1) of adult offspring, we used a general linear model and a Poisson distribution (Neter et al., 2004). The models had three fixed factors: temperature treatment (3 levels), location (3 levels), and sex (2 levels), and we included all interactions among temperature treatment, location, and sex. For the model evaluating percent body fat, we included two covariates—offspring size (pronotum width) and brood size. For the models evaluating fluctuating asymmetry, we included brood size as a covariate. Raw data exhibited normally distributed residuals and fit the assumptions of the model. We included the ID number of the brood as a random effect in these models because we measured two individuals from each brood. We used Proc MIXED in SAS for the analysis (SAS 9.3 SAS Institute).

## 3 RESULTS

### 3.1 Parental reproductive performance

#### 3.1.1 Carcass preparation and reproductive success

**FIGURE 4:**
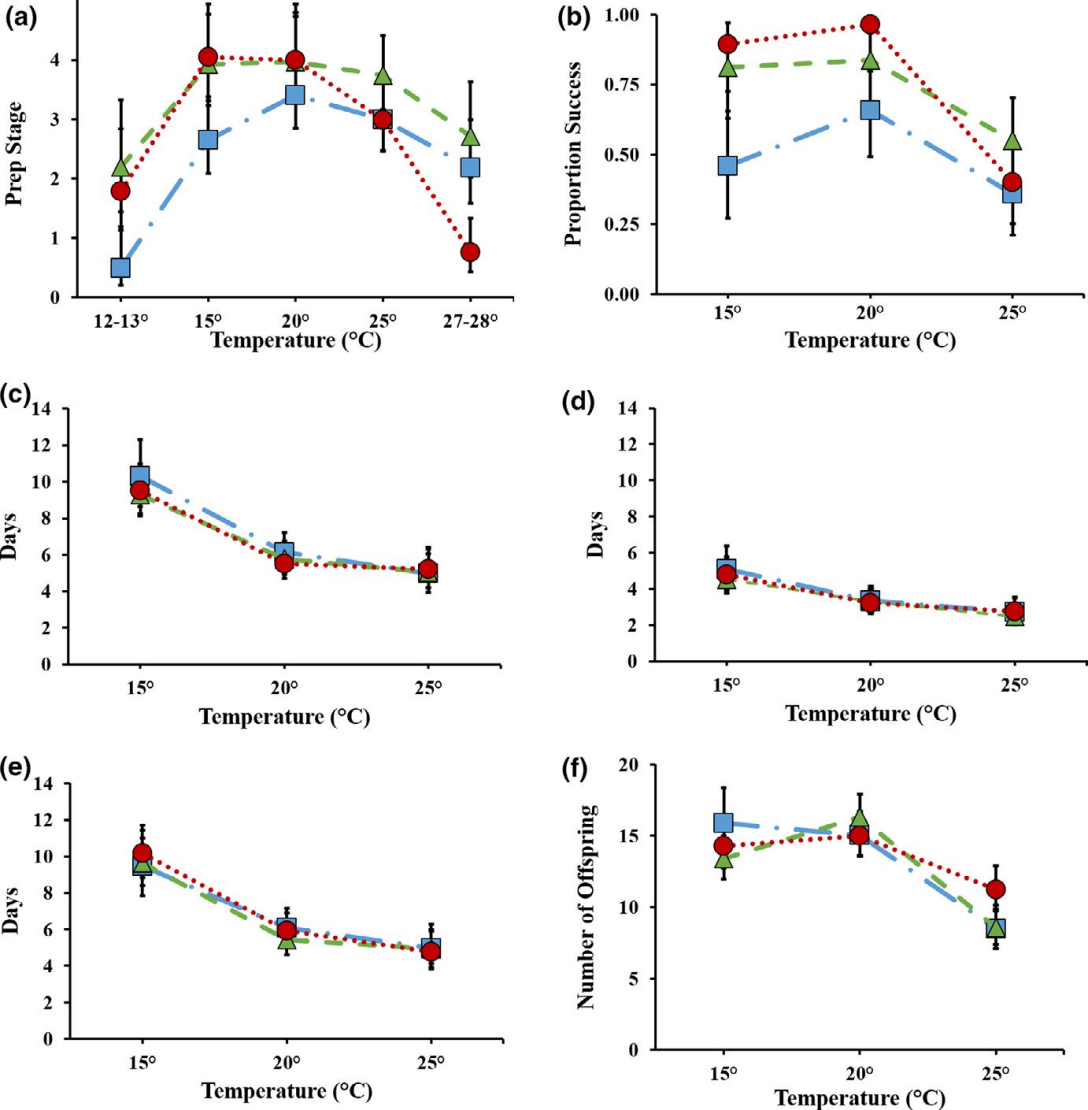
(a) Mean stage of final carcass preparation (±95% CI) at five temperature treatments (high latitude population 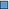
—long dash‐dot line; medium latitude population 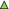
—dashed line; and low latitude population 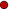
—dotted line); we scored the stage of final carcass preparation using the ethogram summarized in Table 2. (b) Mean proportion of broods that resulted in viable offspring (±95% CI) at three temperature treatments. (c) Mean number of days (±95% CI) of brood hatching asynchrony at three temperature treatments. We calculated hatching asynchrony as the spread of hatching dates within a brood (i.e., the number of days between the first and last arriving first instar larvae on the prepared carcass). (d) Mean number of days (±95% CI) until carcass is fully prepared at three temperature treatments. (e) Mean number of days (±95% CI) before larval dispersal at three temperature treatments. (f) Mean number of offspring (±95% CI) produced at three temperature treatments

The degree of carcass preparation was significantly affected by temperature, location of origin, and the interaction between temperature and location, but it was not affected by male or female mass (Table 3). All three populations had a reduction in their level of carcass preparation at both high‐ and low‐temperature extremes (Figure 4a). On average, HL beetles prepared carcasses less than ML and LL populations at the coolest temperature. Conversely, LL beetles prepared carcasses less than HL and ML beetles at the highest temperature but prepared carcasses further at the coolest temperature. This pattern is consistent with a local adaptation pattern in the willingness to initiate reproduction at marginal temperatures.

**TABLE 3:** ANCOVA table for degree of carcass preparation. Bold values are statistically significant

Response variable	Effect	*df*	*χ* ^2^	*p*‐Value
Final prep stage	Temperature	4	91.45	**˂.0001**
	Location	2	22.56	**<.0001**
	Temperature × Location	8	33.30	**˂.0001**
	Female Mass	1	0.26	.6070
	Male Mass	1	0.08	.7738
	Deviance	346	0.65	1

All three populations reproduced successfully only within a relatively narrow temperature range. Across populations, however, there was considerable variation in their response to temperature with only some of the traits we measured adaptively responding to selection from latitudinally based temperature variation. The probability of producing offspring (i.e., reproductive success) was significantly affected by temperature, location, and the temperature by location interaction, but was not affected by male or female size (Table 4). Populations experienced differing degrees of reproductive success at the three temperatures. At both 15° and 20°C, LL and ML beetles had similar and higher overall reproductive success; however, between 20° and 25°C, a crossing reaction norm was observed between LL and ML populations where LL populations experienced a sharper decrease in success at the highest temperature (Figure 4b). At both 15° and 20°C, HL beetles experienced the lowest rates of success; however, success rate was similar to LL populations at 25°C (Figure 4b). In part, these patterns support both hypotheses: the pattern expressed among populations across all three temperatures is somewhat consistent with a counter‐gradient variation expectation where high temperatures are the selective stressful factor. In contrast, the crossing reaction norms exhibited by the LL and ML populations are consistent with local adaptation.

**TABLE 4:** ANCOVA table for response variables characterizing adult reproductive performance. Bold values are statistically significant

Response variable	Effect	*df*	*χ* ^2^	*p*‐Value
Proportion of successful broods	Temperature	2	40.26	**˂.0001**
	Location	2	20.96	**<.0001**
	Temperature × Location	4	9.75	**.0448**
	Female Mass	1	1.61	.2051
	Male Mass	1	0.19	.6640
Number of offspring	Temperature	2	99.57	**˂.0001**
	Location	2	2.87	.2385
	Temperature × Location	4	13.52	**.0090**
	Female Mass	1	8.80	**.0030**
	Male Mass	1	1.98	.1590
Days to prepare carcass	Temperature	2	43.81	**˂.0001**
	Location	2	0.70	.7057
	Temperature × Location	4	0.42	.9806
	Female Mass	1	1.33	.2486
	Male Mass	1	0.04	.8327
Days before dispersal	Temperature	2	98.39	**˂.0001**
	Location	2	0.28	.8690
	Temperature × Location	4	1.04	.9043
	Female Mass	1	0.12	.7242
	Male Mass	1	0.08	.7715
Hatching asynchrony	Temperature	2	66.85	**˂.0001**
	Location	2	1.02	.5999
	Temperature × Location	4	2.06	.7245
	Female Mass	1	4.25	**.0393**
	Brood Size	1	4.76	**.0291**

#### 3.1.2 Hatching asynchrony

Hatching asynchrony was significantly affected by temperature treatment, and female mass and brood size (Table 4). Overall, there is greater asynchrony at the lowest temperatures and less asynchrony at the highest temperature (Figure 4c). Both female mass and brood size are negatively related to asynchrony, with large broods being more synchronous than small broods and larger females producing more synchronous broods. This pattern is not consistent with any adaptive expectations.

#### 3.1.3 Parental developmental timelines and reproductive output

The three populations differed on how temperature affected the likelihood of producing adult offspring. The LL and ML populations consistently performed better across all three temperatures than the HL population, suggesting that high temperatures are a strong selective force. This result is consistent with counter‐gradient variation. The crossing reaction norms of the ML and LL populations are consistent with a local adaptation hypothesis, but in the opposite direction—local maladaptation. The LL population had a lower success rate than the ML population at the warmest temperature (Figure 4b). This crossing reaction norm does not support an adaptive explanation.

Developmental timing represented as either number of days for carcass preparation, or number of days to dispersal of larvae was significantly affected by temperature (Table 4). Developmental timelines among all three populations across all temperatures responded in a similar way with timelines being extended at lower temperatures and shortened at higher ones (Figure 4c–e). This pattern is not consistent with any adaptive expectation.

The number of offspring produced (i.e., final brood size) was significantly affected by temperature, the temperature by location interaction, and female mass (Table 5). Relative to the other populations, the HL population produces the largest brood size at 15°C, the ML population produces the largest brood size at 20°C, and the LL population produces the largest brood size at 25°C. Overall, populations experience no differences in brood size between 15°C and 20°C, but all three populations were lower at 25°C (Figure 4f). This pattern is consistent with the local adaptation expectation.

**TABLE 5:** ANCOVA table for response variables characterizing offspring performance. Bold values are statistically significant

Response variable	Effect	*df* num/den	*F*‐value	*p*‐Value
Larval growth rate	Temperature	2/159	56.63	**˂.0001**
	Location	2/159	0.19	.8232
	Temperature × Location	4/159	0.73	.5743
	Offspring Mass	1/159	3.81	.0526
	Brood Size	1/159	19.36	**˂.0001**
Adult offspring mass	Temperature	2/148	40.41	**˂.0001**
	Location	2/148	2.75	.0671
	Temperature × Location	4/148	0.88	.4802
	Female Mass	1/148	0.028	.6003
	Male Mass	1/148	0.01	.9408
	Brood Size	1/148	89.95	**˂.0001**
Percentage body fat	Temperature	2/161	3.43	**˂.0348**
	Location	2/155	2.48	.0867
	Temperature × Location	4/154	0.96	.4309
	Sex	1/165	0.23	.6298
	Temperature × Sex	2/163	2.80	.0636
	Location × Sex	2/165	0.08	.9204
	Temperature × Location × Sex	4/162	0.23	.9210
	Offspring Pronotum Width	1/303	19.66	**˂.0001**
	Final Brood Size	1/183	17.95	**˂.0001**

### 3.2 Offspring performance

#### 3.2.1 Offspring growth rate and body size

**FIGURE 5:**
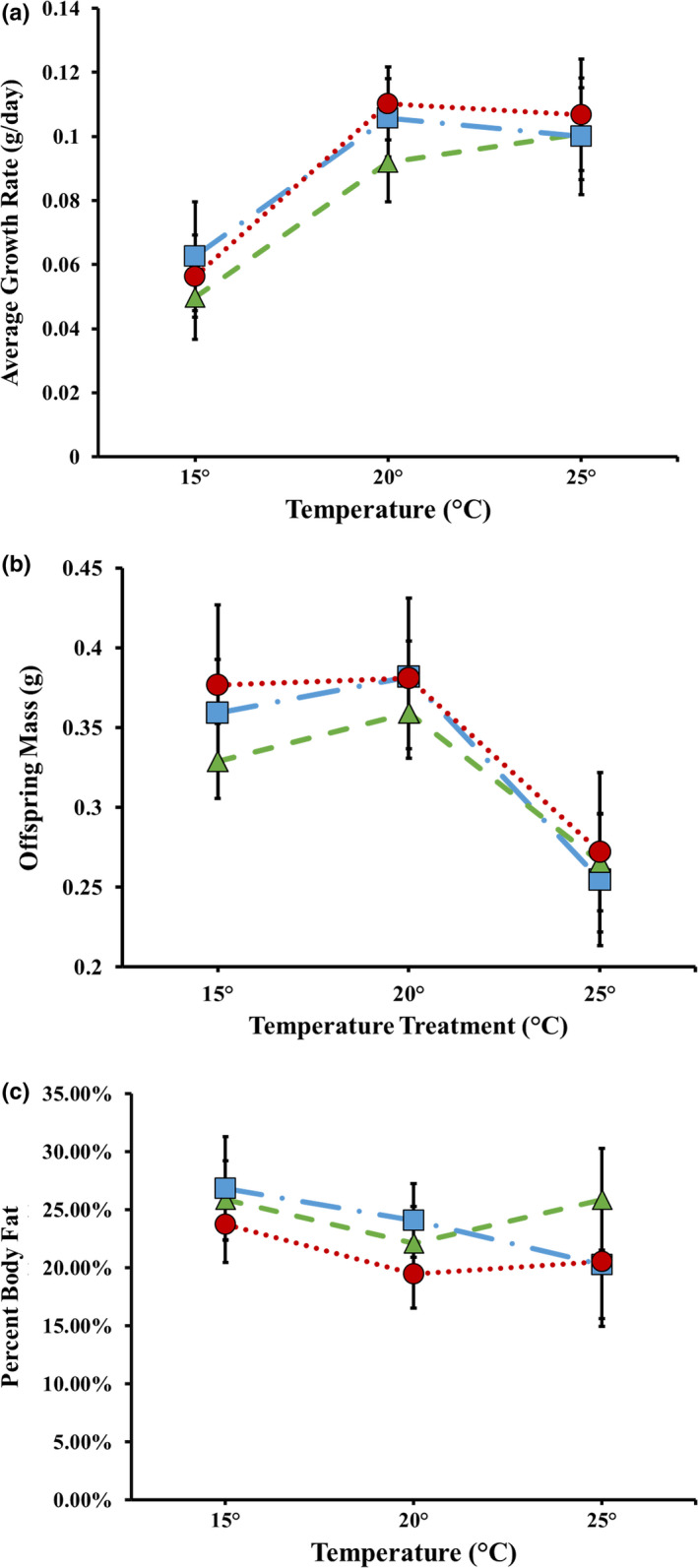
Mean offspring performance measurements (±95% CI) at three temperature treatments for three populations (high latitude population 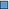
—long dash‐dot line; medium latitude population 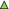
—dashed line; and low latitude population 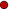
—dotted line); (a) larval growth rate (g/day); (b) offspring mass (g); and (c) percent body fat

Larval growth rate was significantly affected by temperature treatment and brood size (Table 5). All three populations exhibited a hump‐shaped response pattern, with the middle temperature being highest for all locations (Figure 5a). Brood size had a negative effect on growth rate. This pattern is not consistent with any adaptive expectation.

Body size of adult offspring was significantly affected by temperature and the covariate, brood size (Table 5). Newly eclosed offspring from all three populations had similar body mass at 15° and 20°C; however, offspring body mass decreased for all populations at 25°C (Figure 5b). Larger brood sizes resulted in smaller offspring mass. This pattern is not consistent with any adaptive expectation.

#### 3.2.2 Offspring developmental stability

Developmental stability, as measured by fluctuating asymmetry in three different positions on the beetles, was not significantly affected by any of the fixed effects, interactions, or the covariate. Developmental stability exhibits no discernable pattern of variation among locations, treatments, or sexes (Table 6). This pattern is not consistent with any adaptive expectation.

**TABLE 6:** ANOVA table for three measures of fluctuating asymmetry. Mean square error (analogous to a residual) is 46,105, 10,732, and 4,022 for the three measures of fluctuating asymmetry respectively

Response variable	Effect	*df* num/den	*F*‐value	*p*‐Value
Fluctuating Asymmetry Position 1	Temperature	2/155	2.48	.0869
	Location	2/155	0.22	.8052
	Temperature × Location	4/155	0.46	.7651
	Sex	1/180	0.00	.9571
	Temperature × Sex	2/176	0.23	.7985
	Location × Sex	2/178	0.81	.4454
	Temperature × Location × Sex	4/175	0.07	.9917
	Final Brood Size	1/155	0.26	.6139
Fluctuating Asymmetry Position 2	Temperature	2/154	0.52	.5971
	Location	2/154	0.29	.7473
	Temperature × Location	4/154	1.02	.3998
	Sex	1/185	0.00	.9697
	Temperature × Sex	2/180	0.27	.7612
	Location × Sex	2/183	0.17	.8478
	Temperature × Location × Sex	4/178	0.21	.9350
	Final Brood Size	1/154	0.71	.4001
Fluctuating Asymmetry Position 3	Temperature	2/154	1.02	.3645
	Location	2/155	2.50	.0851
	Temperature × Location	4/155	0.75	.5611
	Sex	1/180	0.24	.6270
	Temperature × Sex	2/176	1.19	.3078
	Location × Sex	2/178	0.31	.7344
	Temperature × Location × Sex	4/174	0.23	.9193
	Final Brood Size	1/155	0.44	.5079

Percent body fat of offspring was significantly affected by temperature treatment, offspring body size, and brood size (Table 5). Across all populations, those raised at 15°C had higher fat content compared to those raised at 20°C or 25°C (Figure 5c). Individuals from larger broods had higher percent body fat when compared to individuals from smaller broods. Furthermore, larger individuals had higher percentage body fat than smaller individuals across broods. This pattern is not consistent with any adaptive expectation.

## 4 DISCUSSION

Because we evaluated multiple traits across multiple temperatures, we were able to uncover surprising levels of complexity in the expression of reproductive traits among populations of *N. orbicollis*. Our results indicate that there is locally adaptive variation among the three populations in the initiation of a breeding attempt at extreme temperatures with beetles being *less likely* to initiate a breeding attempt when exposed to a temperature extreme they are most likely to encounter in their local environment (Figure 4a,b). Rather than selection on the ability to perform at temperature extremes per se*,* we show that there is selection on avoiding investing in a costly reproductive attempt that is likely to fail, that is, they are sensitive to the ecological constraint they are most likely to encounter. Second, our results show that there is locally adaptive variation in brood sizes across the three temperatures. Although there was not a significant population effect, there was a significant interaction effect with each population producing the largest brood at the temperature they were most likely to encounter in their home environment. All three populations produced smaller broods at the warmest temperature, suggesting that warmer temperatures are an important constraint across the range of *N. orbicollis*. These unique findings provide evidence that *N. orbicollis* responds to temperature cues when initiating a reproductive attempt, and how much they adjust their broods’ size depends on temperature in ways specific to their source location.

We found no differences among populations in the amount of time necessary for adults to prepare a carcass or to produce offspring (Table 4). Furthermore, we found no differences among populations in measures of offspring performance (Table 5). Why is there no variation in the reproductive timelines and life history traits measured across the three populations? One reason may be the narrow range of temperatures within which reproduction is restricted. Strong selection on avoiding unsuccessful reproductive attempts at extreme temperatures may preclude selection from shaping other aspects of reproductive behavior at extreme temperatures such as the time necessary to reproduce successfully. The result is a lack of covariation among the suite of reproductive traits downstream from the initiation of a reproductive attempt. Behavioral constraints on downstream selection have been demonstrated in *Sceloporus* and *Anolis* lizards, where strong selection on behavioral thermoregulation has limited adaptations in physiological and structural traits (Buckley, 2015; Muñoz & Losos, 2018).

Only temperature affected developmental timeline length. Similar results have been found in other insects including the corn leafhopper *D. maidis* (Van Nieuwenhove et al., 2016) as well as other ectotherms (Välimäki, Kivelä, Mäenpää, & Tammaru, 2012; Van Wingerden, Musters, & Maaskamp, 1991). Why is *N. orbicollis* restricted to a narrow range of temperatures when reproducing, especially when reproductive opportunities may be limited given the relative rarity of small vertebrate carcasses (Eggert & Müller, 1997; Hanski & Cambefort, 1991)? At lower temperatures, the costs of the extended period of parental care and longer development time of the offspring may outweigh any potential fitness benefit. At higher temperatures, development time was much faster but increased social immunity costs associated with higher bacterial loads may constrain reproduction at higher temperatures (Cotter et al., 2010). Temperature may mediate a trade‐off between reproducing while conditions are favorable, and the beetles’ ability to balance lifetime reproductive success. Total reproductive output could remain constant among populations, but the typical trade‐off between offspring size and number could be observed among populations with one population producing more offspring (at the expense of offspring size) and the other producing larger offspring (at the expense of numbers).

Previous research has indicated that populations of ectotherms that inhabit higher latitudes often have broader thermal tolerances including a substantial warm tolerance and ability to withstand cold exposure when compared to populations from lower latitudes (Lancaster, 2016; Lancaster, Dudaniec, Hansson, & Svensson, 2015). However, this does not seem to be the case for reproduction in *N. orbicollis*. The HL population achieved the lowest levels of reproductive success across all temperatures (Figure 4b). This may be a result of stronger selection on the two lower latitude populations in managing extreme temperatures (i.e., counter‐gradient variation). Specifically, the HL population has more days within the upper and lower thermal constraints observed in this experiment than do the lower latitude populations (ncdc.noaa.gov accessed on 9/10/2013). As a result, the HL population has an extended breeding season compared to the other populations, potentially reducing selection to breed successfully at extreme temperatures.

Studies predict that North America will become warmer under current climate models (Garris, Mitchell, Fraser, & Barrett, 2015). As a result, burying beetle populations will experience temperatures that are not currently experienced in their source range. Increases in temperature could affect burying beetles in two ways. First, it would decrease the number of days in a season within the range of amenable temperatures. Second, it would create a mismatch between the mechanism (i.e., HL avoids cool days but now it is hotter) and the new temperature regime. The sister species of *N. orbicollis*, the endangered, federally listed, American burying beetle (*Nicrophorus americanus* Olivier), may be disproportionately affected by this mismatch because its current range is in three isolated populations in Oklahoma‐Arkansas, Nebraska, and Rhode Island (Lomolino, Creighton, Schnell, & Certain, 1995). Without adequate gene flow, these populations may not be able to adapt quickly enough to rapidly changing environmental conditions. However, additional research is needed to evaluate the effect of temperature on *N. americanus* reproduction.

Understanding the interactions between the environment and an organisms’ life history is necessary to understand behavioral patterns and potential mechanisms responsible for variation in reproductive behavior along latitudinal gradients (Parsons & Joern, 2014; Välimäki et al., 2012; Van Nieuwenhove et al., 2016). A latitudinal gradient in reproductive behavior has resulted in variation in the ability to initiate reproduction and reproduce successfully at temperature extremes in *N. orbicollis*. We show that rather than selection to maximize performance at temperature extremes per se, selection on a behavioral mechanism which depends on reliable environmental cues influences whether or not beetles attempt breeding. Once beetles initiated reproduction, each population regulated brood size in a manner that maximized the number of offspring at temperatures that were more likely to be experienced in their source range. The influence of temperature on offspring survival is not known and provides an important avenue for future exploration.

## AUTHOR CONTRIBUTIONS

**Brandon M. Quinby:** Conceptualization (equal); Data curation (equal); Formal analysis (equal); Funding acquisition (equal); Investigation (equal); Methodology (equal); Visualization (equal); Writing‐original draft (lead); Writing‐review & editing (equal). **Mark C. Belk:** Data curation (equal); Formal analysis (equal); Writing‐review & editing (equal). **J. Curtis Creighton:** Conceptualization (equal); Data curation (equal); Funding acquisition (equal); Investigation (equal); Methodology (equal); Project administration (lead); Resources (lead); Writing‐original draft (supporting); Writing‐review & editing (equal).

## PERMITS

We conducted this research under USFWS permit TE61124B‐0 issued to J. C. Creighton.

## Data Availability

All data used in this study have been deposited at Dryad, Dataset: https://doi.org/10.5061/dryad.hhmgqnkdd
